# Skin Microbiota and Pathological Scars: A Bidirectional Two‐Sample Mendelian Randomization Study

**DOI:** 10.1111/jocd.16720

**Published:** 2024-12-09

**Authors:** Ying Huang, Qinghua Yang

**Affiliations:** ^1^ Department of General Plastic Surgery Plastic Surgery Hospital, Chinese Academy of Medical Sciences and Peking Union Medical College Beijing People's Republic of China

**Keywords:** hypertrophic scars, keloid, Mendelian randomization, skin microbiota

## Abstract

**Background:**

Pathological scars (PSs), resulting from abnormal skin repair, chronic inflammation, and fibrosis, affect millions of people. Previous studies have demonstrated that skin microbiota (SM) plays a role in cutaneous inflammation and healing, but the interplay between PSs and SM remains unclear yet.

**Objective:**

To investigate the causal associations between SM and two specific PSs: hypertrophic scars (HSs) and keloids.

**Methods:**

A bidirectional two‐sample mendelian randomization (MR) analysis using genetic data for SM, HS, and keloids was conducted. The random‐effects inverse variance weighted (IVW) method was used as the primary approach, along with multiple MR methods. False discovery rate (FDR) correction was employed to address multiple testing.

**Results:**

In forward analysis, the family *Moraxellaceae* and order *Pseudomonadales* exhibited the same significant protective effects on keloids (odds ratio [OR]: 0.849, 95% confidence interval [CI]: 0.770–0.935, *q*2 = 0.03626). The class *Betaproteobacteria* (OR: 0.938, 95% CI: 0.894–0.985, *q*1 = 0.01965) and genus *Bacteroides* (OR: 0.928, 95% CI: 0.884–0.973, *q*1 = 0.00889) each demonstrated a suggestive protective effect on HSs and keloids, respectively. Some limited evidence suggested that order *Actinomycetales* contributes to an increased risk of keloids. In reverse analysis, keloids were found to have negative effects on the class *Gammaproteobacteria* with limited evidence. There was no detectable evidence of horizontal pleiotropy or heterogeneity.

**Conclusion:**

This study provided evidence for the causalities between SM and PSs, which laid foundation for furthering clinical practice and research of microorganism–skin interaction.

**Abbreviations**
ASV: amplicon sequence variants
CI: confidence interval
FDR: false discovery rate
GWAS: genome‐wide association study
HS: hypertrophic scar
IV: instrumental variable
IVW: inverse variance weighted
MR: Mendelian randomization
OR: odds ratio
PSs: pathological scars
*q*1: *p*‐value after FDR correction at the corresponding feature level
*q*2: *p*‐value after FDR correction at the global level
SM: skin microbiota
SNP: single nucleotide polymorphism
WM: weighted median

## 1 Introduction

Skin wound healing is a highly intricate process, which involves the coordinated induction of inflammation and tissue repair. Certain circumstances, such as surgical procedures, accidental injuries, burns, and dermal diseases with injuries reaching the reticular dermis, can disrupt this process, leading to the development of pathological scars (PSs) [1]. Although there is no specific record on the prevalence of scars, it should not be overestimated considering the global annual volume of surgeries estimated at around 310 million [2]. Hypertrophic scars (HSs) and keloids are two pathophysiological distinct subtypes of abnormal scars that not only impose physical discomfort, cosmetic problems, and deformities but also psychological burdens on patients, significantly impairing their life quality [3]. While HSs tend to confine themselves within the boundaries of the original wound and often resolve spontaneously, keloids extend beyond the initial wound borders unchecked [4]. The pathophysiology of PSs remains incompletely understood; however, unrelenting and persistent inflammatory responses are believed to contribute to the excessive proliferation and accumulation of scar tissue [1, 5]. Various factors contribute predisposition to scar formation, including genetic factors such as chromosomal alterations [6], single nucleotide polymorphisms (SNPs) [7, 8], and epigenetic modifications [9, 10]. Additionally, systemic risk factors such as elevated sex hormones [11] and comorbidities of chronic inflammatory conditions (e.g., rheumatism [12] and Castleman disease [1]), along with local factors such as re‐wounding [1], infections [5], anatomical locations [13], and high tension [14, 15], have been found to accelerate scar formation. Despite a plethora of prevention strategies and treatment algorithms that have been proposed for PSs, their effectiveness remains inconsistent, particularly when considering variations in anatomical sites and population distribution [3, 11, 16].

In recent years, interest in skin microbiota (SM) has grown due to advancements in 16S rRNA gene sequencing techniques [17]. As the largest organ in the human body, the skin serves as a dynamic interface for millions of bacteria, fungi, and viruses [18]. Host–microbe interactions occur not only on the extensive epithelial surface but also within the underlying dermis and dermal adipose tissue [19]. It is now evident that these microorganisms play a pivotal role in maintaining host homeostasis and overall health, including the wound healing, repairing, and inflammatory disorders of the skin itself [20, 21, 22, 23, 24, 25]. Furthermore, disruptions in the SM have been observed in the context of PSs [26, 27].

**FIGURE 1:**
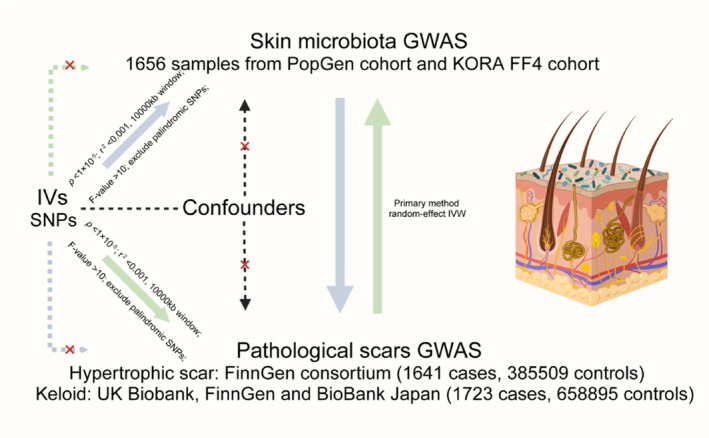
Overview of the bidirectional Mendelian randomization study. GWAS, genome‐wide association study; IVs, instrumental variables; IVW, inverse variance weighted; SNPs, single nucleotide polymorphisms.

Despite these findings, the causal association between SM and PSs remains unexplored. To address this gap, the present study employed Mendelian randomization (MR), a method leveraging genetic variants based on Mendel's law, to assess causality [28]. MR analysis serves as a natural randomized controlled trial, circumventing ethical constraints and high costs. By utilizing large‐scale genome‐wide association study (GWAS) data, this bidirectional two‐sample MR aimed to (i) identify specific microbiome signatures associated with PSs and (ii) investigate the reciprocal relationships between PSs and variations in the skin microbiome (Figure 1).

## 2 Materials and Methods

### 2.1 Study Design

This two‐sample MR analysis was conducted based on three core assumptions: (1) relevance assumption: the genetic variants are associated with the SM; (2) independence assumption: the genetic variants share no unmeasured cause with pathologic scars; (3) exclusion restriction assumption: the genetic variants do not affect outcomes except through their potential effect on the exposures [29]. SNPs, as the most common genetic variants, were applied as instrumental variables to proxy for exposure. In step one, forward MR analysis was employed to investigate the causal relationship between the SM and pathologic scars. In step two, reverse MR analysis was performed to examine whether genetic liability to PSs influences the levels of SM. The reporting of this study adheres to the Strengthening the Reporting of Observational Studies in Epidemiology Using Mendelian Randomization (STROBE‐MR) guideline [30].

### 2.2 Instrument Variable Search and Selection

Summary‐level genetic data from GWAS were primarily derived from European and East Asian populations. Ethnic approvals can be obtained in each data source.

For SM, genetic instruments were retrieved from a meta‐analysis of two adult German cohorts, the PopGen and KORA FF4 (https://www.ebi.ac.uk/gwas/, GCST90133164‐GCST90133313) [31]. A total of 1656 skin samples collected from different skin sites (e.g., antecubital fossa, retroauricular fold, forehead, and dorsal and volar forearms) were analyzed for multivariate community composition (beta diversity) and bacterial taxa, including 3 phyla, 4 classes, 7 orders, 7 families, 15 genera, and 43 ASVs.

SNPs associated with HS were obtained from the latest version of data released by the FinnGen consortium (R10, L12_HYPETROPHICSCAR) [32]. The FinnGen study represents a significant genomics initiative, involving the analysis of an extensive collection of more than 500 000 Finnish biobank samples. A total of 1641 HS cases recruited based on diagnostic criteria of International Classification of Disease (ICD)‐8, ‐9, and ‐10 standards, as well as 385 509 controls, were included in this study.

GWAS data for keloids were acquired from a cross‐population meta‐analysis that showed no significant heterogeneity among the UK Biobank, FinnGen, and BioBank Japan [33]. The study included 668 cases of European ancestry diagnosed according to ICD‐10 criteria, alongside 481 224 controls [32, 34]. Besides, 1055 cases of East Asian ancestry diagnosed by collaborating physicians at affiliated hospitals and 177 671 controls from BioBank Japan, a nationwide hospital‐based prospective cohort, were included [35].

Given that these datasets represent the most recent and extensive collections and exhibit no overlap between the exposure and the outcome datasets, we established a significance threshold of *p* < 1 × 10^−5^, consistent with the previous study [36], to yield a relatively large number of IVs.

Linkage disequilibrium (LD) clumping was applied with an *r* ^2^ threshold of 0.001 and a distance cutoff of 10 000 kb to guarantee the independence of SNPs. In addition, IVs with *F*‐values ≤ 10 were categorized as weak instruments. Palindromic SNPs were removed during the harmonization of summary‐level exposure and outcome data.

### 2.3 Mendelian Randomization Analyses

Two‐sample MR analyses and additional meta‐analysis were conducted by TwoSampleMR, meta packages in R (version 4.3.1) [37]. The random‐effects inverse variance weighted (IVW) was primarily used to examine the relationship of SM and HS. The IVW method combines individual IV estimates by weighting them according to the inverse of their variances with requirement that all of the genetic variants involved are valid IVs [38]. To confirm the causal inference, we performed additional analyses using the weighted median (WM) approach. The WM method estimates by taking a WM of individual IV estimates and provides valid estimates even when up to 50% of the IVs are invalid [39]. Moreover, if the direction of any fitted line from complementary analysis methods (e.g., MR‐Egger, weighted mode and simple mode) in the scatter plot contradicts that of the IVW method, further investigation will be conducted to explore potential sources of heterogeneity or violations of assumptions. To mitigate the challenges posed by multiple significance testing, we employed false discovery rate (FDR) correction on the *p*‐values derived from the IVW analysis [40]. Corrections were conducted at both the classification level (*q*1) and the global level (*q*2). Associations with a corrected *q*2 value below 0.05 were considered statistically significant, and those with *q*1 < 0.05 < *q*2 were regarded as suggestive associations. Several statistic methods were employed to assess potential violations of the necessary IV assumptions. For pleiotropy assessment, the MR‐Egger intercept test [41] and MR pleiotropy residual sum and outlier (MR‐PRESSO) global test were conducted [42]. Horizontal pleiotropy was considered present if either test yielded a statistically significant result (*p* < 0.05). The MR‐PRESSO outlier test was also used to identify and correct for horizontal pleiotropic outliers, if any were detected [42]. Cochran's IVW *Q* test was conducted to detect heterogeneity at a 5% significance level [43]. Additionally, leave‐one‐out sensitivity analysis was performed to assess the contribution of each individual IV [42].

## 3 Results

### 3.1 Overview of Forward MR Analysis

**FIGURE 2:**
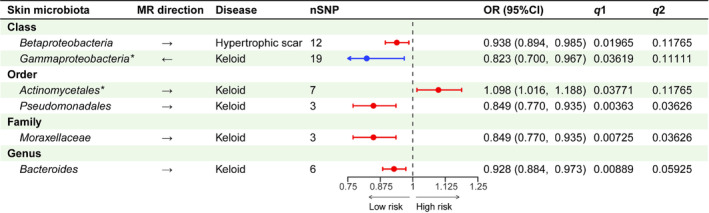
Forest plot of forward and reverse MR analysis. The significant associations identified through IVW analysis, which remain stable even after classification‐level FDR correction (*q*1 < 0.05) is presented. The arrows in the 2nd column symbolize the direction of MR analysis, with “→” indicating a forward MR analysis where the skin microbiota is considered the cause and pathological scars as the effect, and vice versa. *p‐value > 0.05 in the WM method. nSNP, number of single nucleotide polymorphisms involved; *q*1, *p*‐value after FDR correction at the corresponding classification level; *q*2, *p*‐value after FDR correction at the global level.

**FIGURE 3:**
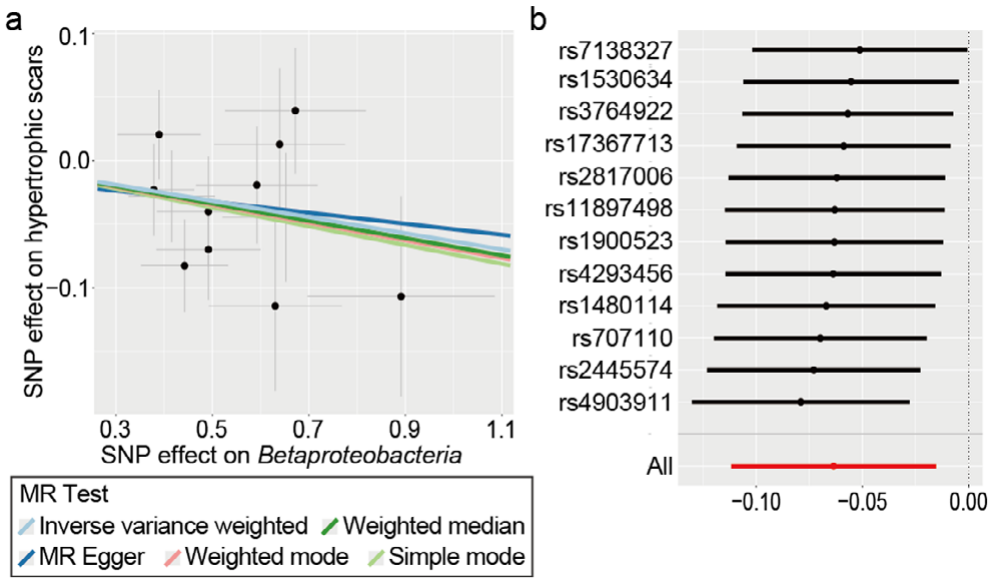
Scatter plot and sensitivity analysis for the association between *Betaproteobacteria* and HSs. (a) Scatter plot of the association; (b) leave‐one‐out sensitivity analysis.

**FIGURE 4:**
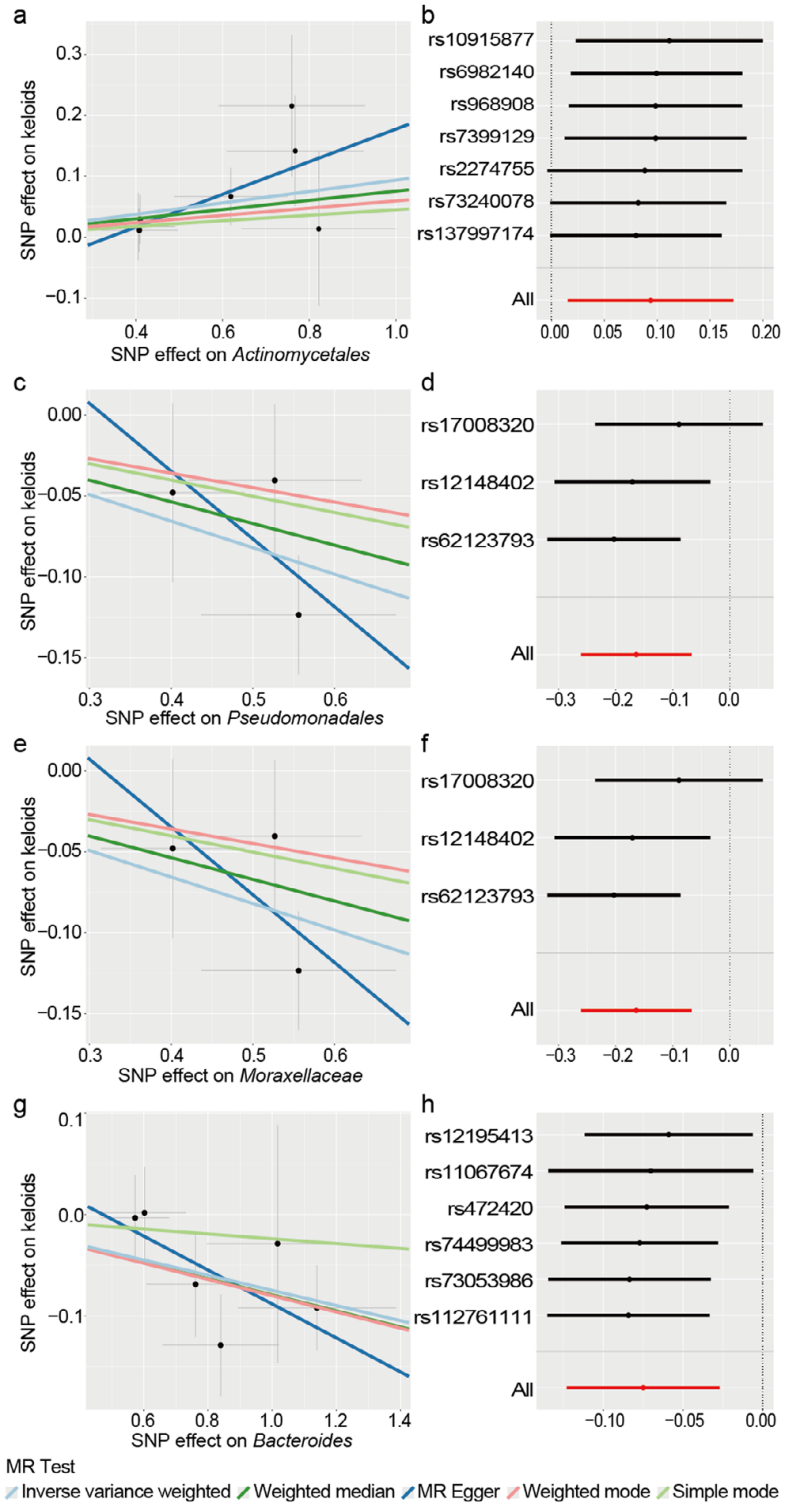
Scatter plot and sensitivity analysis for the associations between SM and keloids. (a) Scatter plot of the association between *Actinomycetales* and keloids; (b) leave‐one‐out sensitivity analysis for the association between *Actinomycetales* and keloids; (c) scatter plot of the association between *Pseudomonadales* and keloids; (d) leave‐one‐out sensitivity analysis for the association between *Pseudomonadales* and keloids; (e) scatter plot of the association between *Moraxellaceae* and keloids; (f) leave‐one‐out sensitivity analysis for the association between *Moraxellaceae* and keloids. (g) scatter plot of the association between *Bacteroides* and keloids; (h) leave‐one‐out sensitivity analysis for the association between *Bacteroides* and keloids.

SNPs associated with SM were identified at various taxonomic levels: beta diversity (*n* = 113), ASV (*n* = 1251), genus (*n* = 401), family (*n* = 186), class (*n* = 151), order (*n* = 229), and phylum (*n* = 92). Initial IVW analysis revealed that 142 SNPs within 19 microbial taxa potentially influence PSs (Table S1). Among these, five suggestive relationships supported by FDR corrections are presented in Figure 2. Horizontal pleiotropy was not detected in the MR‐Egger intercept test and MR‐PRESSO analyses (Table S2). No heterogeneity among the screened SNPs was observed according to Cochrane's *Q* test. As expected, the leave‐one‐out analysis did not find significant alteration of the estimated results after removing individual SNPs in turn (Figures 3 and 4 and Figures S1 and S2).

#### 3.1.1 Causal Effects of SM on HSs

Among the seven microbial taxa identified by the IVW method, the class *Betaproteobacteria* in the antecubital fossa exhibited a significant protective role (odds ratio [OR]: 0.938, 95% confidence interval [CI]: 0.894–0.985) against HSs. This was confirmed through the consistent direction of multiple methods, namely, the WM method (*p* = 0.04593) and classification‐level FDR correction (*q*1 = 0.01965) (Figures 2 and 3). However, the significance decreased after global correction (*q*2 = 0.11765).

#### 3.1.2 Causal Effects of SM on Keloids

The order *Pseudomonadales* (OR: 0.849, 95% CI: 0.770–0.935, *q*1 = 0.00363), family *Moraxellaceae* (OR: 0.849, 95% CI: 0.770–0.935, *q*1 = 0.00725), and genus *Bacteroides* (OR: 0.928, 95% CI: 0.884–0.973, *q*1 = 0.00889) in antecubital fossa were associated with a suggestive low risk among the 12 potential findings. The WM method consistently yielded estimates that fortified these associations. The scatterplot concurrently unveiled the harmonious directionality of the supplemental methods (Figure 4). Upon incorporating more stringent FDR corrections on a global scale, the initial two results maintained stability (*q*2 = 0.03626 for both).

On the other hand, the order *Actinomycetales* from forehead showed limited evidence of involvement in keloid development (OR: 1.098, 95% CI: 1.016–1.188, *q*1 = 0.03771), as supporting results from WM method and global correction were not obtained.

### 3.2 Reverse MR Analysis

**FIGURE 5:**
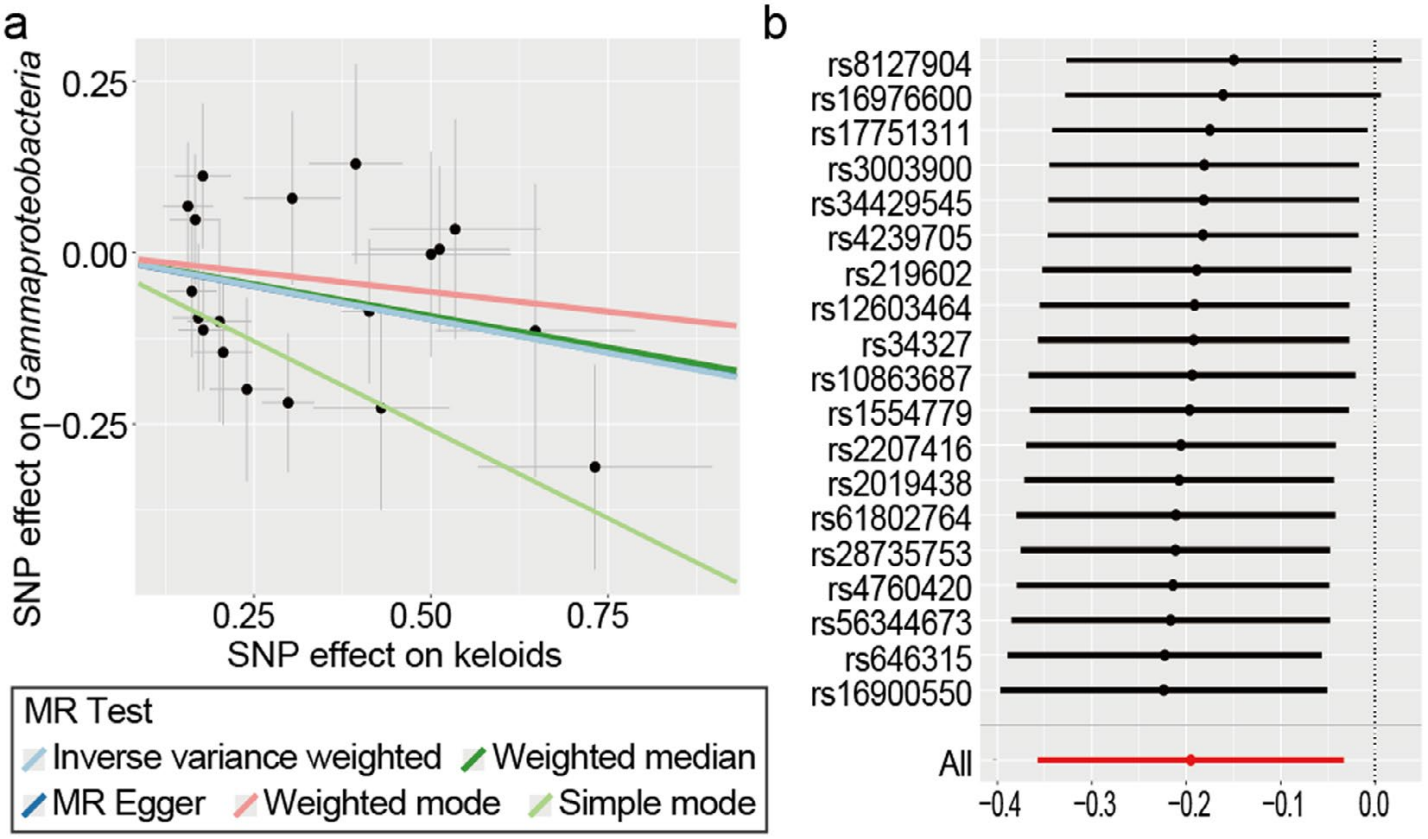
Scatter plot and sensitivity analysis for the association between keloids and *Gammaproteobacteria*. (a) Scatter plot of the association; (b) leave‐one‐out sensitivity analysis.

95 SNPs associated with HS and 41 SNPs associated with keloids were discovered. Respectively, 35 and 18 SNPs were associated with SM (Table S3). As for HS, 15 potential associations with SM were identified. The estimated effects of HS on the family *Streptococcaceae* and genus *Streptococcus* varied significantly between the PopGen and KORA FF4 cohorts. Additional meta‐analyses using random‐effects inverse‐variance model revealed no significant association (the family *Streptococcaceae*: OR 1.053, 95% CI: 0.571–1.535, *I* ^2^ = 90.3%; genus *Streptococcus*: OR 1.013, 95% CI: 0.598–1.428, *I* ^2^ = 88.2%) (Figure S3 and Table S4). Given the contradictory findings and substantial heterogeneity, we subjected the remaining 11 associations to FDR corrections, excluding the four. None of the results reached statistical significance. Regarding keloids, a suggestive negative effect on the class *Gammaproteobacteria* (OR: 0.823, 95% CI: 0.700–0.967, *q*1 = 0.03619) in the forehead emerged from the seven potential relationships identified in the IVW method. Figure 5 supports for the causal hypothesis, demonstrating a consistent trend in the fitted line. Nevertheless, the evidence was limited, as the results did not maintain statistical significance when applying the WM method (*p* = 0.12145) and global FDR correction (*q*2 = 0.11111). The statistical analyses revealed no evidence of heterogeneity or horizontal pleiotropy (Figures S4 and S5).

## 4 Discussion

The development of PSs was influenced by the interplay between intrinsic and extrinsic factors, as well as variations in skin microbiome composition [3, 44]. This bidirectional two‐sample MR study enhances our knowledge of the complex dialogue between pathological conditions and a vital component of the skin. The identification of varying levels of evidence indicating the protective or risk effects of the SM on HSs and keloids marked the first examination of such findings. Notably, keloids also exhibited a suggestive negative effect on specific microbes.

*Pseudomonadota*, formerly known as *Proteobacteriota*, comprises five classes: *Alphaproteobacteria*, *Betaproteobacteria*, *Deltaproteobacteria*, *Epsilonproteobacteria*, and *Gammaproteobacteria*. *Pseudomonadota* is highly prevalent among skin bacteria and represents a significant portion of the opportunistic microbiome [22, 45]. Microbial DNA of *Pseudomonadota*, particularly *Betaproteobacteria* and *Gammaproteobacteria* exhibits widespread distribution in the epidermis, follicles, dermis, and adipose tissue, indicating a close and direct interaction with human tissue [19]. Contrary to the established positive correlations between the abundance of gut *Proteobacteria* and inflammation [46], our study corroborated the findings of Zhou et al. [22] which demonstrated a predominantly negative association between skin *Proteobacteria* and cytokine levels. Specifically, our results indicated that class *Betaproteobacteria*, order *Pseudomonadales*, and family *Moraxellaceae* mitigated the risk of PSs, while keloids were negatively associated with class *Gammaproteobacteria*.

*Betaproteobacteria* is a commonly found class on various skin sites, especially in dry and moist areas [45]. *Betaproteobacteria* in antecubital fossa showed a suggestive role in preventing the development of HSs, but no individual subordinate member (e.g., order *Burkholderiales* and family *Neisseriaceae*) demonstrated statistically significant associations with HSs. Considering that *Betaproteobacteria* is a diverse group consisting of over 75 genera and 400 species, the overall association may be driven by the other members or synergistic effects with other taxa within the complex microbial networks [47]. In order to harness the existing evidence, there arises an immediate necessity for a sequencing and profiling method that is compatible with SM.

*Gammaproteobacteria* is the most phylogenetically and physiologically diverse class within the *Pseudomonadota* and encompasses various medically significant bacteria [48]. This group comprises facultatively anaerobic and fermentative bacteria. In the present study, the estimated effect sizes for the associations between keloids and both order *Pseudomonadales* and family *Moraxellaceae* in the antecubital fossa are identical, indicating a protective role of these bacteria in keloid development. A thorough examination of the data revealed that the two taxa were linked to three coincident SNPs with varying degrees of correlation (i.e., *p*‐value, beta, and standard error). Despite the fact that the family *Moraxellaceae* is classified under the order *Moraxellales* in the reconstructed taxonomic framework of the class, the original study employed the Ribosomal Database Project v.16 release for taxonomic classification [36, 49]. Therefore, both the GWAS data and results make sense, as *Moraxellaceae* used to be the only identified family within the order *Pseudomonadales*. The adverse effect on keloid formation might be attributed to the anti‐inflammation role of cutaneous *Gammaproteobacteria*. This group of bacteria has been observed to negatively correlate with proinflammatory cytokines while positively correlating with increased regulatory T cell frequencies [22, 50]. Further analysis of bacterial lysates may help unveil the underlying mechanisms, as related taxa have recently demonstrated a consistent anti‐inflammatory effect in a skin wound model [51]. Furthermore, *Acinetobacter*, which is the largest and sole genus within the family *Moraxellaceae*, did not show a significant association with PSs. None of the multiple ASVs investigated did, either. This implied that the low‐abundance bacteria within *Moraxellaceae* may genuinely act as protectors against keloid formation. On the contrary, the reverse MR analysis revealed a noteworthy finding: keloids exert a negative effect on *Gammaproteobacteria* at the class level. The tumor‐like growth of keloids is accompanied by damage to local cutaneous appendages (e.g., sebaceous glands, sweat glands, and hair follicles) as well as metabolism [3, 52]. The chemistry of skin niche plays a pivotal role in shaping its microbiome composition [53]. Except for aberrantly upregulation of arachidonic acid, increasing interleukin (IL)‐1α, IL‐1β, IL‐6, and tumor necrosis factor‐α have also been reported [1, 54]. Such disturbance to the skin niche may adversely affect *Gammaproteobacteria*, which preferentially thrive in dry areas with low levels of proinflammatory factors [22, 45, 50]. The identification of these mutual associations underscored the intricate ecology underlying keloid development.

*Bacteroides* is a gram‐negative, anaerobic, rod‐shaped genus within phyla *Bacteroidota* and is one of the predominant bacteria in the human digestive tract and skin [55]. *Bacteroides* produces inflammation‐inhibiting compounds such as short‐chain fatty acids and secondary bile acids [56, 57]. Besides, *Bacteroides* is among the few known microbes involved in tryptophan metabolism, which produces tryptophan catabolites that play a crucial role in immune responses and homeostasis [58, 59]. For example, indole‐3‐aldehyde has been shown to negatively regulate skin inflammation in patients with atopic dermatitis [60]. The aforementioned anti‐inflammatory effects may elucidate the potential safeguarding function of *Bacteroides* in the process of keloid formation. However, the underlying mechanism requires exploration from various perspectives, such as by refining the classification of suspected bacteria at a finer level and metabolome analyses of local interstitial fluid.

*Actinomycetales*, commonly called *Actinomycetes*, is a gram‐positive and primarily aerobic order within the phylum *Actinomycetota* [61]. The bacterial group accounts for approximately two‐thirds of all known antibiotics, yet little is known about its role as an abundant skin microflora [45, 62]. Our results indicated that *Actinomycetales* on the forehead promote keloid development. Meanwhile, no similar effect is identified in any other bacteria (e.g., *Corynebacterium* and *Propionibacterium*) within the same phylum. The host–microbiome interaction may mediate scar formation via promoting fibrosis and inflammation since recent research, indicating a positive correlation between *Actinomyces* and epidermal growth factor and a negative correlation with IL‐7 [22].

*Streptococcus* is a genus within the family *Streptococcaceae*, comprising gram‐positive bacteria that lack endospores. Certain species can be highly pathogenic (e.g., *Streptococcus pyogenes* [63]), while some others are recognized as evaluated sources in clinical medicine (e.g., *S. thermophilus* exhibit anti‐fibrotic and antioxidant properties [64, 65]). The non‐significant synthesized estimations of inconsistent results from the two cohorts could be attributed to complex interactions among genetic effects, specific *Streptococcaceae* species, and environmental influences [53]. Further research focusing on geographical variations following HSs and the functions of these bacteria could deepen our understanding of the pathological process.

Nonetheless, there are some limitations. Firstly, a total of 1656 skin swabs from three different microenvironments (i.e., dry, moist, and sebaceous) of healthy European adults contributed to the most recent and largest GWAS datasets for SM [31]. But characterization of these microbiomes might have limitations when considering PSs or issues related to microbial carriage sites. Further investigation across diverse populations, including various races (e.g., Black people) and age groups (e.g., adolescents), is necessary for generalizability of current findings, especially when accounting for ethnic differences and hormonal fluctuations that influence both microbial remodeling and scar formation [11, 66]. Besides, samples from typical skin sites with a high prevalence of scars (e.g., keloids on the chest, scapular region, lower jaw, and earlobe) are essential [3]. It is because bacteria manifest significant niche specificity and function distinctly across body sites [22, 45]. Secondly, to identify instruments for the two‐sample MR without overlap, employing a relatively liberal *p*‐value threshold (*p* < 1 × 10^−5^), despite validation through F‐statistics and sensitivity analyses, may bias causal estimates toward the null due to potential risk of weak instruments [67, 68, 69]. Extensive whole‐genome sequencing and microbial profiling are warranted to verify our conservative results, including the contentious links between scars and specific microorganisms (e.g., *Streptococcaceae* and *Streptococcus*), as well as to detect additional crucial microbiota at finer taxonomic levels (e.g., ASV). Rigorous clinical trials and experimental verification in the future are needed as well. Thirdly, this study aimed to discuss the general associations between scars and bacterial composition but could hardly answer changes in bacterial metabolic activities or distribution using static classification methods employed in current diagnosis, sampling, and sequencing approaches. Detailed categorizations of scars at different phases, multi‐time‐point sampling, and metabolite profiling of key strains based on the dynamic characteristics of both PSs and SM are necessary [3, 22]. Moreover, while our current study focuses on cutaneous bacteria as an entry point into this field, a comprehensive understanding of the deeper complexities requires the inclusion of other components within the skin microbial ecosystem (e.g., phages, viruses, and fungi).

## 5 Conclusions

This study highlights the mutually exclusive connections between SM (i.e., *Moraxellaceae*, *Betaproteobacteria*, *Gammaproteobacteria*, *Bacteroides*) and PSs, as well as the involvement of *Actinomycetales* in keloid development. From a clinical standpoint, identifying key microbial taxa offers potential to develop innovative therapeutic approaches and biomarkers for disease prediction and monitoring. Modifying the composition or function of the SM in specific phases and microenvironments using personalized strategies, including healthy behaviors, probiotics, prebiotics, and microbiome transplants, could be a promising approach to modulate scar formation and progression [70]. For scientific research, current findings suggest that a focus on SMs will aid in elucidating the mechanisms of scar formation. Nevertheless, future investigations involving larger populations and multidimensional considerations are required to deepen and widen the knowledge of complex relationship between scars and our coexisting microbial communities.

## Author Contributions

Conceptualization: Q.Y. and Y.H.; data curation: Y.H.; formal analysis: Y.H.; investigation: Y.H.; methodology: Q.Y. and Y.H.; project administration: Q.Y. and Y.H.; software: Y.H.; supervision: Q.Y.; visualization: Y.H.; roles/writing – original draft: Y.H.; and writing – review and editing: Q.Y. and Y.H.

## Ethics Statement

The authors confirm that the ethical policies of the journal, as noted on the journal's author guidelines page, have been adhered to. No ethical approval was required, as this is a review article with no original research data.

## Conflicts of Interest

The authors declare no conflicts of interest.

## Supporting information


**FIGURE S1.** Leave‐one‐out sensitivity analysis for the associations between SM and HSs. Dots and bar (estimated effect and 95% CI) in black represent MR results obtained after excluding the corresponding SNP on the y‐axis, and red ones represent the overall MR results. (a) *Micrococcaceae*; (b) *Rhodobacteraceae*; (c) *Anaerococcus (unc.) (ASV007)*; (d) *Enhydrobacter (unc.) (ASV016)*; (e) *Novosphingobium (unc.) (ASV063)*; (f) *Propionibacterium acnes (ASV001)*.

**FIGURE S2.** Leave‐one‐out sensitivity analysis for the association between SM and keloids. Dots and bar (estimated effect and 95% CI) in black represent MR results obtained after excluding the corresponding SNP on the y‐axis, and red ones represent the overall MR results. (a) *Proteobacteria*; (b) *Flavobacteriaceae*; (c) *Haemophilus*; (d) *Diaphorobacter nitroreducens (ASV008)*; (e) *Micrococcus (unc.) (ASV021)*; (f) *Novosphingobium (unc.) (ASV063)*; (g) *Paracoccus (unc.) (ASV072)*; (h) *Propionibacterium acnes (ASV001)*.

**FIGURE S3.** Meta‐analysis for associations between two controversial taxa and HSs. The findings from the KORA FF4 and PopGen cohorts exhibit conflicting results regarding the impact of HSs on the family *Streptococcaceae* and genus *Streptococcus*. Consequently, the pooled results show no statistical significance and a considerable level of heterogeneity.

**FIGURE S4.** Leave‐one‐out sensitivity analysis for the association between HSs and SM. Dots and bar (estimated effect and 95% CI) in black represent MR results obtained after excluding the corresponding SNP on the y‐axis, and red ones represent the overall MR results. (a) *Lactobacillales*; (b) *Chryseobacterium*; (c) *Actinomycetales (unc.) (ASV015)*; (d) *Cloacibacterium (unc.) (ASV045)*; (e) *Corynebacterium (unc.) (ASV004)*; (f) *Novosphingobium (unc.) (ASV063)*; (g) *Paracoccus (unc.) (ASV054)*; (h) *Staphylococcus (unc.) (ASV070)*; (i) *Staphylococcus (unc.) (ASV114)*; (j) *Staphylococcus epidermidis (ASV013)*; (k) *Streptococcus salivarius (ASV022)*.

**FIGURE S5.** Leave‐one‐out sensitivity analysis for the association between keloids and SM. Dots and bar (estimated effect and 95% CI) in black represent MR results obtained after excluding the corresponding SNP on the y‐axis, and red ones represent the overall MR results. (a) *Paracoccus*; (b) *Anaerococcus (unc.) (ASV007)*; (c) *Corynebacterium (unc.) (ASV004)*; (d) *Diaphorobacter (unc.) (ASV035)*; (e) *Micrococcus (unc.) (ASV021)*; (f) *Paracoccus (unc.) (ASV054)*.

**Table S1.**.

## Data Availability

Datasets related to this article can be found at https://www.ebi.ac.uk/gwas/ and https://www.finngen.fi/en/access_results, two open‐source online data repositories hosted at genome‐wide association study data.
