# Supplementary figures and images for: Skin Microbiota and Pathological Scars: A Bidirectional Two‐Sample Mendelian Randomization Study
Source: J Cosmet Dermatol. 2024 Dec 9;24(2):e16720. doi: 10.1111/jocd.16720 (PMC11845961; doi:10.1111/jocd.16720)

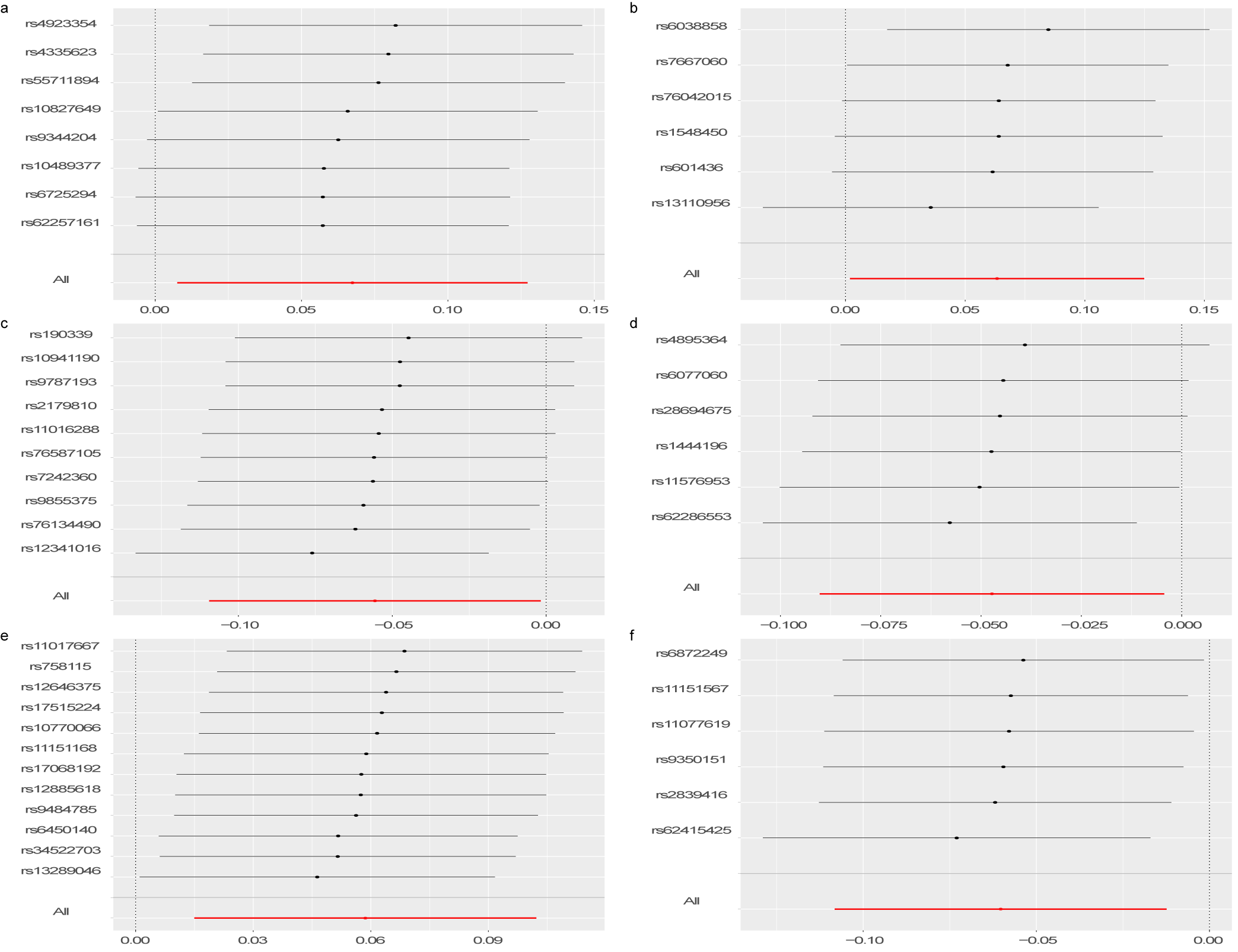

Supplement: Supplementary file 1 — FIGURE S1. Leave‐one‐out sensitivity analysis for the associations between SM and HSs. Dots and bar (estimated effect and 95% CI) in black represent MR results obtained after excluding the corresponding SNP on the y‐axis, and red ones represent the overall MR results. (a) Micrococcaceae; (b) Rhodobacteraceae; (c) Anaerococcus (unc.) (ASV007); (d) Enhydrobacter (unc.) (ASV016); (e) Novosphingobium (unc.) (ASV063); (f) Propionibacterium acnes (ASV001). [file JOCD-24-e16720-s006.tif]

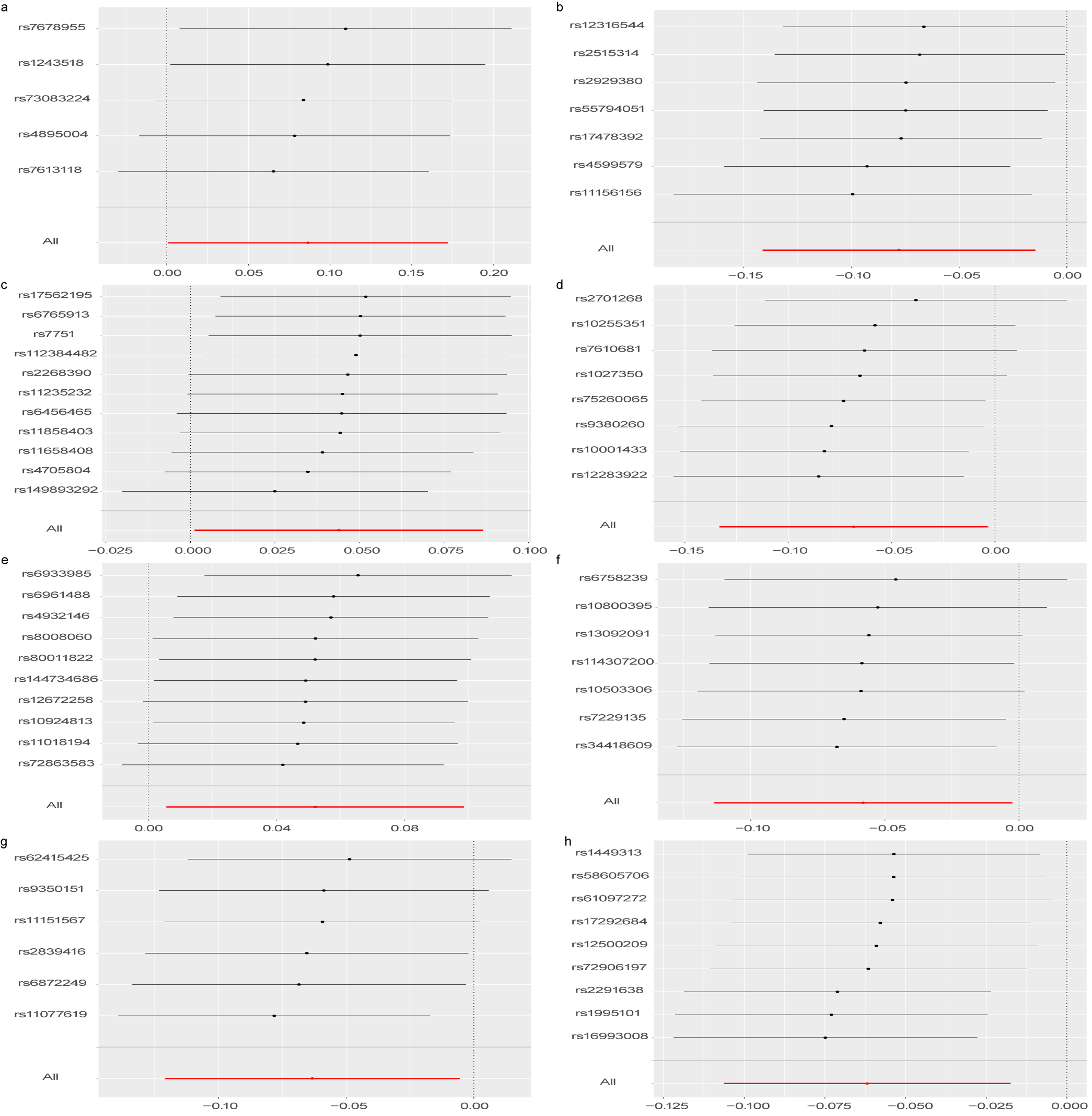

Supplement: Supplementary file 2 — FIGURE S2. Leave‐one‐out sensitivity analysis for the association between SM and keloids. Dots and bar (estimated effect and 95% CI) in black represent MR results obtained after excluding the corresponding SNP on the y‐axis, and red ones represent the overall MR results. (a) Proteobacteria; (b) Flavobacteriaceae; (c) Haemophilus; (d) Diaphorobacter nitroreducens (ASV008); (e) Micrococcus (unc.) (ASV021); (f) Novosphingobium (unc.) (ASV063); (g) Paracoccus (unc.) (ASV072); (h) Propionibacterium acnes (ASV001). [file JOCD-24-e16720-s001.tif]

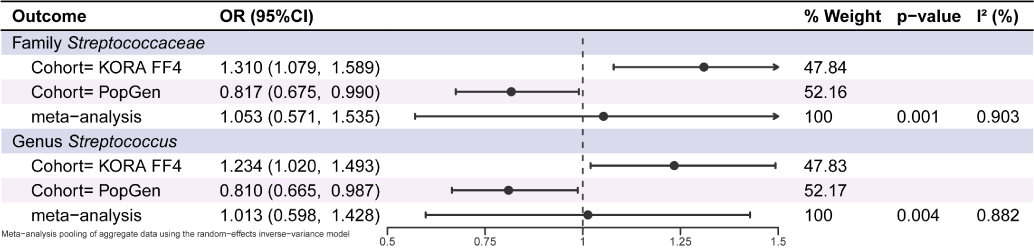

Supplement: Supplementary file 3 — FIGURE S3. Meta‐analysis for associations between two controversial taxa and HSs. The findings from the KORA FF4 and PopGen cohorts exhibit conflicting results regarding the impact of HSs on the family Streptococcaceae and genus Streptococcus. Consequently, the pooled results show no statistical significance and a considerable level of heterogeneity. [file JOCD-24-e16720-s004.tif]

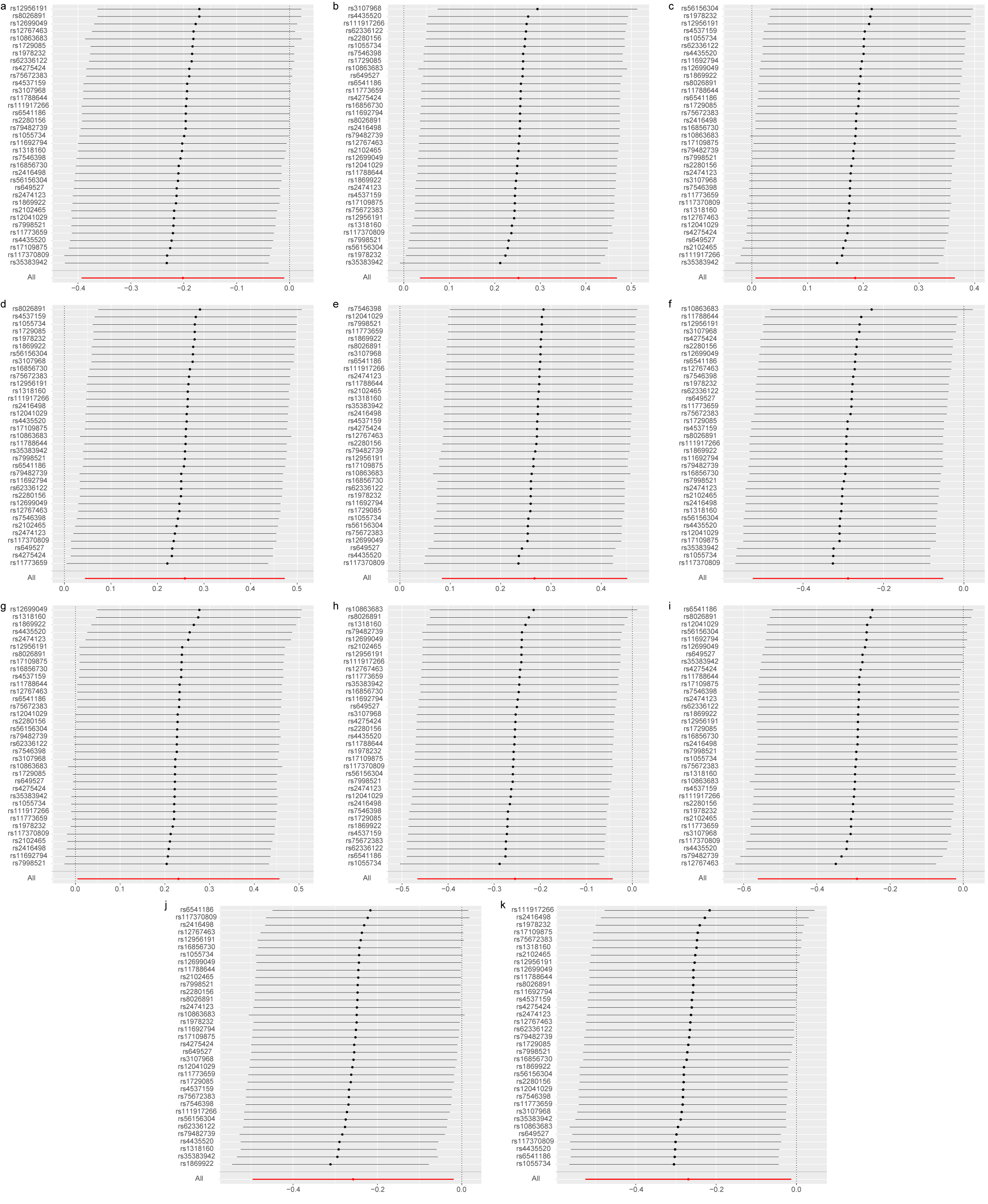

Supplement: Supplementary file 4 — FIGURE S4. Leave‐one‐out sensitivity analysis for the association between HSs and SM. Dots and bar (estimated effect and 95% CI) in black represent MR results obtained after excluding the corresponding SNP on the y‐axis, and red ones represent the overall MR results. (a) Lactobacillales; (b) Chryseobacterium; (c) Actinomycetales (unc.) (ASV015); (d) Cloacibacterium (unc.) (ASV045); (e) Corynebacterium (unc.) (ASV004); (f) Novosphingobium (unc.) (ASV063); (g) Paracoccus (unc.) (ASV054); (h) Staphylococcus (unc.) (ASV070); (i) Staphylococcus (unc.) (ASV114); (j) Staphylococcus epidermidis (ASV013); (k) Streptococcus salivarius (ASV022). [file JOCD-24-e16720-s005.tif]

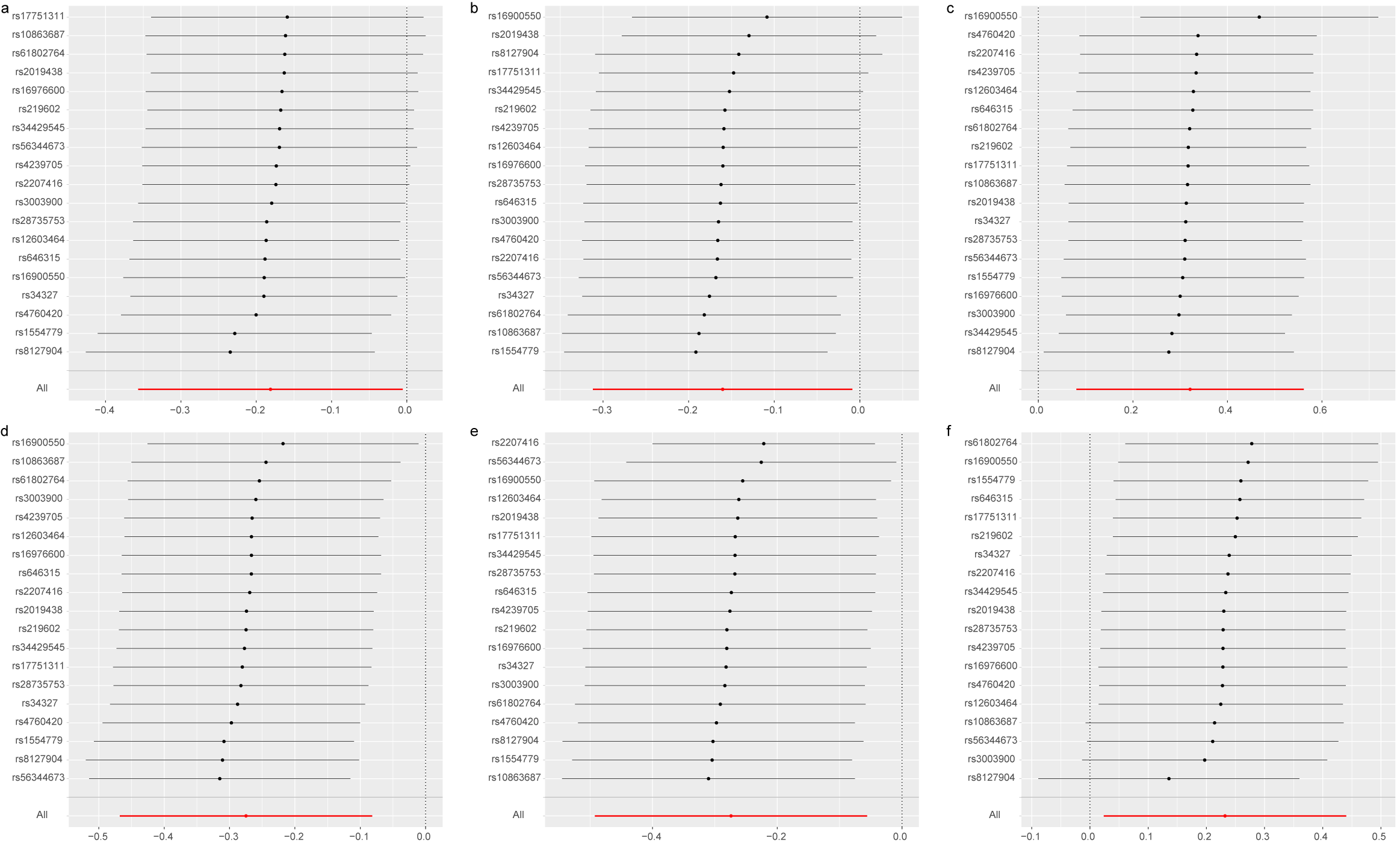

Supplement: Supplementary file 5 — FIGURE S5. Leave‐one‐out sensitivity analysis for the association between keloids and SM. Dots and bar (estimated effect and 95% CI) in black represent MR results obtained after excluding the corresponding SNP on the y‐axis, and red ones represent the overall MR results. (a) Paracoccus; (b) Anaerococcus (unc.) (ASV007); (c) Corynebacterium (unc.) (ASV004); (d) Diaphorobacter (unc.) (ASV035); (e) Micrococcus (unc.) (ASV021); (f) Paracoccus (unc.) (ASV054). [file JOCD-24-e16720-s003.tif]
